# Reduced Left Ventricular Ejection Fraction as a Marker of Vulnerability to Healthcare-Associated Infections in Coronary Care Unit Patients: A Single-Centre Cohort Study

**DOI:** 10.3390/jcm15051789

**Published:** 2026-02-27

**Authors:** Daniela-Mirela Vîrtosu, Angela Dragomir, Simina Crișan, Silvia Luca, Oana Pătru, Ruxandra-Maria Băghină, Mihai-Andrei Lazăr, Alina-Ramona Cozlac, Stela Iurciuc, Constantin-Tudor Luca

**Affiliations:** 1 Doctoral School, “Victor Babes” University of Medicine and Pharmacy, 300041 Timisoara, Romania; daniela.cozma@umft.ro (D.-M.V.); silvia.luca@umft.ro (S.L.); oana.patru@umft.ro (O.P.); ruxandra.croicu@umft.ro (R.-M.B.); 2 Institute of Cardiovascular Diseases Timisoara, 13A Gheorghe Adam Street, 300310 Timisoara, Romania; angela.dragomir@umft.ro (A.D.); lazar.mihai@umft.ro (M.-A.L.); alina-ramona.cozlac@umft.ro (A.-R.C.); constantin.luca@umft.ro (C.-T.L.); 3 Research Center of the Institute of Cardiovascular Diseases Timisoara, 13A Gheorghe Adam Street, 300310 Timisoara, Romania; 4 Cardiology Department, “Victor Babes” University of Medicine and Pharmacy, 2 Eftimie Murgu Sq., 300041 Timisoara, Romania; iurciuc.stela@umft.ro

**Keywords:** left ventricular ejection fraction, healthcare-associated infections, coronary care unit, heart failure, invasive devices, infection prevention, risk stratification, nursing care, device-associated infections, critical cardiac care

## Abstract

**Background/Objectives:** Healthcare-associated infections (HAIs) remain an important cause of morbidity in coronary care units (CCUs). Although left ventricular ejection fraction (LVEF) is central to cardiovascular risk stratification, its relationship with infection susceptibility in CCU patients is poorly defined. We explored the association between LVEF and HAI incidence in a real-world CCU population. **Methods:** We performed a retrospective cohort study including 870 consecutive adult patients admitted to a tertiary CCU. Patients were stratified by LVEF into reduced (<40%) and preserved or mildly reduced (≥40%) groups. HAIs were defined using Centers for Disease Control and Prevention/National Healthcare Safety Network (CDC/NHSN) criteria and required microbiological confirmation. Demographic data, comorbidities, exposure to invasive devices, colonization status and clinical outcomes were collected. Associations with HAIs were assessed using univariate and exploratory multivariable logistic regression. **Results:** Of the 870 patients, 235 (27.0%) had LVEF < 40%. The overall HAI incidence was 1.8% (16/870) and was significantly higher in patients with reduced LVEF compared with those with LVEF ≥ 40% (3.82% vs. 1.10%, *p* = 0.018). Patients with LVEF < 40% had greater exposure to invasive devices (OR 2.06, 95% CI 1.52–2.79, *p* < 0.001). The excess HAI burden was mainly driven by urinary tract infections (1.70% vs. 0.15%, *p* = 0.021). Colonization rates at admission were similar between groups. In univariate analysis, reduced LVEF was associated with higher HAI occurrence, but it did not remain independently associated after adjustment. Admission infection, malignancy, CPAP use, and CCU length of stay ≥5 days emerged as independent factors in the exploratory multivariable model (Nagelkerke R^2^ = 0.247). **Conclusions:** Reduced LVEF is associated with higher HAI incidence in CCU patients, reflecting greater clinical severity, longer hospitalization, and increased exposure to invasive devices. Although not an independent predictor, LVEF appears to function as a clinically useful marker of vulnerability that may support early risk stratification and targeted infection-prevention strategies in CCU settings.

## 1. Introduction

Healthcare-associated infections (HAIs) remain a major cause of morbidity, mortality, and prolonged hospitalization in critically ill cardiac patients. Although coronary care units (CCUs) are primarily dedicated to the management of acute cardiovascular emergencies, patients admitted to these units often present with complex clinical profiles characterized by advanced age, multiple comorbidities, hemodynamic instability, and extensive exposure to invasive monitoring and therapeutic procedures. These features place CCU patients at particularly high risk for HAIs, making infection prevention a central component of quality of care in contemporary cardiovascular medicine [1,2].

Left ventricular ejection fraction (LVEF) is a cornerstone parameter of cardiac systolic function and a well-established predictor of prognosis, clinical instability, and adverse outcomes across a broad spectrum of cardiovascular diseases. Reduced LVEF is associated with impaired tissue perfusion, venous congestion, endothelial dysfunction and neurohormonal activation, mechanisms that may compromise immune defense and increase susceptibility to infection. Moreover, patients with reduced LVEF frequently exhibit a higher burden of comorbidities such as diabetes, chronic kidney disease or obesity, all of which are independently linked to increased infection risk [3,4,5]. From a clinical perspective, these patients usually require more intensive monitoring and a greater number of invasive procedures, including central venous catheters, arterial lines, mechanical ventilation, or urinary catheters, which are well-recognized contributors to HAIs [2].

Despite the central role of LVEF in cardiovascular risk stratification, its potential association with susceptibility to HAIs in CCU patients remains insufficiently explored. Most studies evaluating HAI risk in critical care focus on device exposure, comorbidity burden, or global severity scores, while parameters of cardiac function are rarely incorporated. It, therefore, remains unclear whether reduced LVEF represents an independent risk factor for HAIs, a marker of increased vulnerability, or simply a surrogate for more aggressive and invasive care [6,7,8].

Clarifying this relationship is clinically relevant. Identifying specific cardiac phenotypes at higher risk of infection may improve early risk stratification, support targeted preventive strategies, and allow more efficient allocation of infection-prevention resources in high-dependency cardiac care. Furthermore, determining whether reduced LVEF is associated with distinct patterns of infection may help refine surveillance priorities and optimize patient management.

The objective of this study was to evaluate the association between LVEF and the incidence of HAIs in a large, real-world cohort of CCU patients and to explore whether reduced LVEF is independently associated with HAIs or rather reflects a marker of clinical vulnerability. We compared HAI occurrence, exposure to invasive devices, colonization patterns, and clinical characteristics between patients with reduced LVEF (<40%) and those with preserved or mildly reduced systolic function (≥40%). By addressing an underexplored dimension of vulnerability in CCU populations, this study aims to provide clinically relevant data to enhance infection risk stratification and support evidence-based prevention strategies in critically ill cardiac patients.

## 2. Materials and Methods

### 2.1. Study Design and Population

This retrospective cohort study was conducted in the CCU of the Institute of Cardiovascular Diseases of Timișoara, a tertiary-care center providing specialized cardiovascular care to a large urban and regional population in Western Romania. All consecutive adult patients admitted to the CCU over a 6-month period (1 May–31 October 2024) were screened for eligibility. The study protocol complied with institutional ethical standards and adhered to the principles of the Declaration of Helsinki.

A total of 870 consecutive patients were included in the final analysis. All adult patients admitted during the study period were considered eligible, irrespective of the presence of infection at admission, as pre-existing infections were recorded separately and were not classified as HAIs. Exclusion criteria were limited to the absence of echocardiographic assessment of LVEF during hospitalization, missing microbiological data, or incomplete medical records preventing reliable outcome evaluation. No additional exclusions were applied in order to preserve the real-world complexity and infection risk profile of CCU patients.

LVEF was assessed by transthoracic echocardiography performed by experienced cardiologists using the biplane Simpson method, based on the first available examination obtained after CCU admission, typically within the first 24 h of hospitalization, in accordance with current international guidelines. Based on LVEF values, patients were stratified into two groups: those with reduced LVEF (<40%), consistent with systolic dysfunction, and those with preserved or mildly reduced systolic function (≥40%).

HAIs were defined according to the Centers for Disease Control and Prevention/National Healthcare Safety Network (CDC/NHSN) criteria as infections not present or incubating at admission and identified at least 48 h after hospital admission. Only microbiologically confirmed infections were considered. Diagnostic sources included positive blood cultures, urine cultures, tracheal or bronchial aspirates, wound or pressure-ulcer cultures, catheter-tip cultures, and Clostridioides difficile toxin assays. Colonization identified through admission screening (nasal, pharyngeal, inguinal, or urinary) was documented separately and was not classified as infection. Admission infection was recorded as a pre-existing infection documented at the time of CCU admission and was treated as a baseline clinical characteristic rather than as a healthcare-associated event. Congestive heart failure was defined as a previously documented clinical diagnosis in the medical records prior to the index CCU admission and was not based on the LVEF measured during the current hospitalization.

Data were extracted from electronic medical records and referred strictly to the CCU hospitalization period. Collected variables included demographic characteristics (age, sex, and environment of origin), clinical data (primary diagnosis, comorbidities such as diabetes, chronic infections, malignancy, and immunosuppression, admission pathway as emergency or non-emergency, and length of stay), and information on invasive procedures. Invasive procedures included peripheral or central venous catheters, arterial lines, urinary catheters, continuous positive airway pressure (CPAP) or endotracheal intubation, temporary or permanent pacemaker leads, and pleural catheters. Microbiological investigations included blood cultures, urine cultures, wound cultures, tracheal aspirates, catheter-tip cultures, stool toxin assays for Clostridioides difficile, and pressure-ulcer sampling when applicable. Screening data consisted of nasal, pharyngeal, inguinal, and urinary colonization assessments performed at admission.

The primary outcome of the study was the occurrence of at least one microbiologically confirmed HAI during CCU hospitalization. Secondary outcomes included the type of HAI, the number of invasive devices per patient, colonization patterns, and length of CCU stay.

### 2.2. Statistical Analysis

All collected data were entered into a Microsoft Excel database, version 2011 (Microsoft Corp., Redmond, WA, USA). Statistical analyses were performed using MedCalc for Windows, version 19.4 (MedCalc Software, Ostend, Belgium) and the Epi Info statistical package, version 7.2.5.0 (Centers for Disease Control and Prevention, Atlanta, GA, USA). Odds ratios (ORs) with 95% confidence intervals (CIs) were calculated. The chi-square test and Fisher’s two-tailed exact test were used, as appropriate according to expected cell counts, to evaluate associations between categorical variables and to compare proportions between groups. A *p*-value < 0.05 was considered statistically significant.

Baseline characteristics were compared between patients with LVEF < 40% and those with LVEF ≥ 40% using the chi-square test or Fisher’s exact test for categorical variables and the Mann–Whitney U test for continuous variables, as appropriate. The incidence of HAIs was calculated for each LVEF group. Results are reported as percentages, medians with interquartile ranges (IQR), or odds ratios (ORs) with 95% confidence intervals (CIs).

To explore whether reduced LVEF remained associated with the occurrence of HAIs after adjustment for major clinical and care-related factors, an exploratory multivariable logistic regression model was constructed. This analysis was not intended to develop or validate a predictive clinical model but rather to identify potential independent associations in a hypothesis-generating framework. Candidate covariates were preselected based on univariate significance and strong clinical plausibility. Given the limited number of events, only a small number of variables were entered simultaneously in the final model to reduce the risk of overfitting. Although the multivariable analysis was exploratory and not intended for clinical prediction model development, a formal calibration assessment was additionally performed using the Hosmer–Lemeshow goodness-of-fit test to enhance methodological robustness and transparency. No external validation or discrimination-focused predictive modeling was undertaken. Because several variables reflected overlapping aspects of clinical severity and intensity of care, potential collinearity was considered when selecting variables for the final model.

## 3. Results

### 3.1. Baseline Characteristics

A total of 870 consecutive patients were included in the analysis. Among them, 235 patients (27.0%) had reduced LVEF (LVEF < 40%), while 635 patients (73.0%) had preserved or mildly reduced systolic function (LVEF ≥ 40%).

Baseline characteristics according to LVEF category are presented in Table 1. There was no significant difference in age between groups (median 65 years (IQR 57.00–74.00) in the LVEF < 40% group vs. 66 years (IQR 54.00–74.00) in the LVEF ≥ 40% group, *p* = 0.75). Sex distribution was also comparable between groups (*p* = 0.170).

Patients with reduced LVEF had a significantly higher prevalence of diabetes mellitus (27.65% vs. 19.52%, *p* = 0.012) and a higher frequency of pre-existing clinically diagnosed congestive heart failure (9.78% vs. 4.40%, *p* < 0.001). The length of CCU stay was significantly longer in the reduced LVEF group (median 5 days, IQR 3.00–8.00) compared with the LVEF ≥40% group (median 4 days, IQR 3.00–5.00; *p* < 0.001).

**Figure 1:**
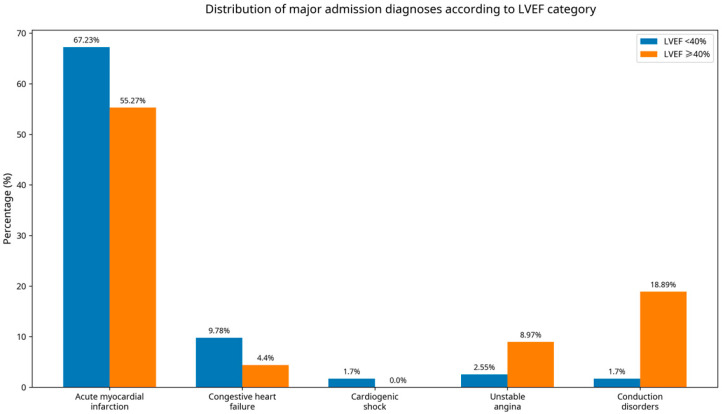
Distribution of major admission diagnoses according to LVEF category. Bars represent the percentage of patients in each diagnostic group with LVEF < 40% and ≥40%, highlighting differences in the clinical profile between reduced and preserved systolic function.

The primary admission diagnoses differed markedly between groups, reflecting distinct clinical phenotypes. Patients with LVEF < 40% were mainly admitted for acute myocardial infarction, congestive heart failure, and cardiogenic shock, indicating a more severe hemodynamic profile, whereas unstable angina and conduction disorders predominated among patients with preserved or mildly reduced LVEF (Figure 1). The complete distribution of admission diagnoses is detailed in Supplementary Table S1.

### 3.2. Invasive Device Exposure

Exposure to invasive devices differed markedly between the two LVEF groups, as seen in Table 2. Patients with LVEF < 40% had significantly higher rates of central venous catheterization (20.85% vs. 3.93%, OR 6.42, 95% CI 3.86–10.69, *p* < 0.001), urinary catheterization (56.17% vs. 26.92%, OR 3.47, 95% CI 2.54–4.74, *p* < 0.001), CPAP use (14.04% vs. 2.20%, OR 7.24, 95% CI 3.80–13.81, *p* < 0.001), endotracheal intubation (22.55% vs. 3.77%, OR 7.41, 95% CI 4.45–12.34, *p* < 0.001), and pleural catheter placement (3.82% vs. 1.10%, OR 3.57, 95% CI 1.31–9.70, *p* < 0.001). Moreover, patients with reduced LVEF were more likely to be exposed to a higher cumulative invasive burden. The probability of being exposed to three or more invasive devices was significantly higher in the LVEF < 40% group compared with the LVEF ≥ 40% group (55.31% vs. 37.48%, OR 2.06, 95% CI 1.52–2.79, *p* < 0.001). Permanent pacemaker implantation was predominantly observed in patients with preserved or mildly reduced LVEF, while it was significantly less common in those with reduced LVEF (18.74% vs. 5.10%, OR 0.23, 95% CI 0.12–0.43, *p* < 0.001).

Colonization screening at admission did not differ significantly between the two groups. Positive colonization at any site was detected in 15.28% of patients with LVEF < 40% and in 17.49% of patients with LVEF ≥ 40% (*p* = 0.468). No statistically significant differences were observed for nasal, pharyngeal, inguinal, or urinary colonization (all *p* > 0.05). Detailed colonization patterns are provided in Supplementary Table S2.

### 3.3. Incidence of HAIs

**Figure 2:**
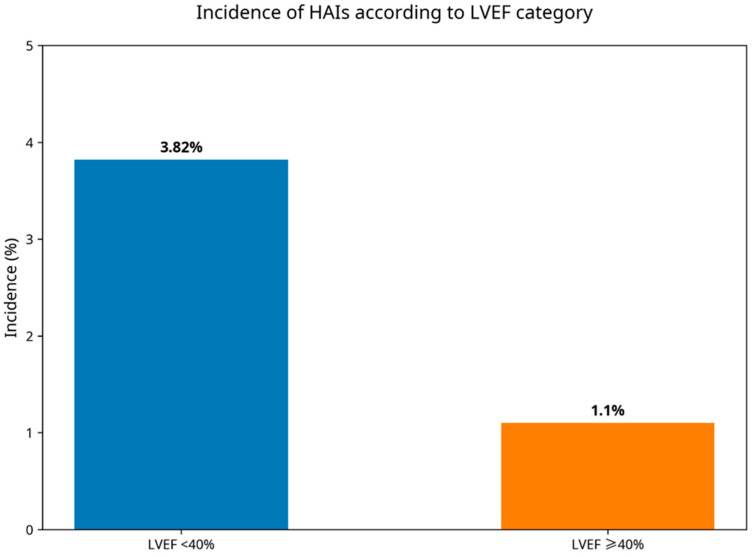
Incidence of HAIs according to left ventricular ejection fraction category. Patients with LVEF < 40% showed a higher incidence of HAIs compared with those with LVEF ≥ 40%.

The overall incidence of HAIs in the cohort was 1.8% (16/870). HAI incidence was significantly higher among patients with reduced LVEF compared with those with preserved or mildly reduced systolic function (3.82% [9/235] vs. 1.10% [7/635], *p* = 0.018). This difference is illustrated in Figure 2.

**Figure 3:**
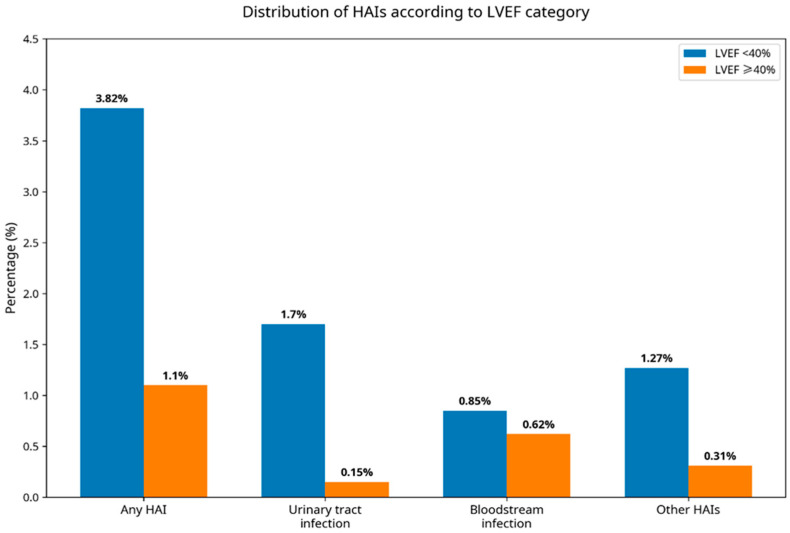
Distribution of HAIs according to LVEF category. The proportion of any HAI, urinary tract infection, bloodstream infection, and other HAIs is consistently higher in patients with LVEF < 40% compared with those with LVEF ≥ 40%, suggesting a higher overall infectious burden in patients with reduced systolic function.

Urinary tract infections were significantly more frequent in the LVEF < 40% group (1.70% vs. 0.15%, *p* = 0.021), whereas no statistically significant differences were observed for bloodstream infections, respiratory infections, catheter-related infections, or Clostridioides difficile infection. The distribution of infection types according to LVEF category is graphically represented in Figure 3.

Median time to HAI onset was 9 days (IQR 6.50–11.50) in patients with LVEF < 40% and 16 days (IQR 4.00–19.00) in patients with LVEF ≥ 40%, *p* = 0.48.

### 3.4. Association Between LVEF and Risk of HAI

In univariate analysis, reduced LVEF was associated with a significantly increased risk of developing a HAI (OR 3.57, 95% CI 1.31–9.70, *p* = 0.018). Other variables significantly associated with HAI risk included admission infection, phlebitis, malignancy, central venous catheter use, urinary catheterization, CPAP use, and a CCU length of stay ≥5 days. Diabetes showed a borderline association with HAI occurrence (*p* = 0.058). Full univariate results are reported in Supplementary Table S3.

**Figure 4:**
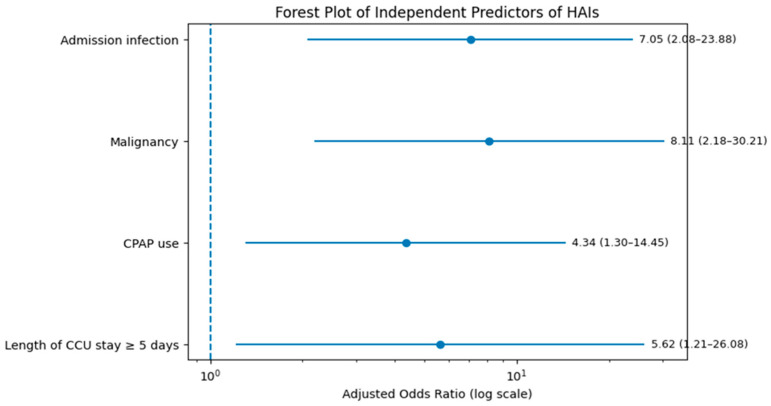
Forest plot showing independent predictors of HAIs identified in the final parsimonious multivariable logistic regression model. Points represent adjusted ORs, and horizontal bars indicate 95% CI. The vertical dashed line marks the null effect (OR = 1).

In multivariable logistic regression analysis, admission infection, malignancy, CPAP use and CCU length of stay ≥5 days remained independent predictors of HAI occurrence (Table 3). The final model explained 24.7% of the variance in HAI occurrence (Nagelkerke R^2^ = 0.247), indicating a moderate explanatory capacity, consistent with the multifactorial nature of HAIs in critically ill cardiac patients. Model calibration analysis indicated acceptable agreement between predicted and observed probabilities (Hosmer–Lemeshow *p* = 0.70). LVEF, diabetes, phlebitis, and central venous catheterization lost statistical significance after adjustment for these variables. The independent predictors and their effect sizes are illustrated in Figure 4.

## 4. Discussion

The present study shows that patients with reduced LVEF (LVEF < 40%) admitted to a CCU have a significantly higher incidence of HAIs and markedly greater exposure to invasive devices. However, after adjustment for clinical severity and care-related factors, LVEF did not remain an independent predictor of HAIs. This finding suggests that reduced LVEF should be interpreted primarily as a marker of vulnerability rather than a direct causal determinant of infection. In other words, systolic dysfunction identifies a subgroup of patients who are clinically more unstable, require more invasive support, have longer hospital stays, and therefore accumulate a higher risk of infection. From an infection-control perspective, LVEF functions as an early warning signal that flags patients in whom preventive strategies should be intensified from the moment of admission.

In our cohort, admission infection, malignancy, CPAP use and a length of CCU stay ≥5 days emerged as variables independently associated with HAI occurence in the exploratory multivariable model. These findings highlight that infection risk in critically ill cardiac patients is driven by a complex interaction between baseline patient vulnerability, disease severity, and intensity of invasive care. Consequently, LVEF should be interpreted as an early clinical signal identifying patients who are more likely to accumulate infection risk through prolonged hospitalization and increased exposure to invasive procedures.

Taken together, our results underscore the clinical value of systolic dysfunction as a readily available marker for targeted infection-prevention strategies in cardiovascular critical care. Integrating cardiac functional status into infection-control protocols may allow earlier risk stratification and more efficient allocation of preventive resources.

### 4.1. Pathophysiological Mechanisms Linking Systolic Dysfunction and Infection Susceptibility

The association between reduced LVEF and increased infection risk is biologically plausible and multifactorial. Growing evidence supports the concept that cardiac dysfunction reflects a complex interplay between structural remodeling, electrical instability, endothelial impairment, and systemic inflammatory activation rather than an isolated mechanical deficit [9]. These alterations profoundly affect immune competence and tissue barrier integrity, creating a biological background of vulnerability that may predispose patients to infectious complications [10]. Reduced cardiac output compromises tissue perfusion and oxygen delivery, impairing neutrophil activity, delaying wound healing, and weakening innate immune responses. In parallel, venous and lymphatic congestion promote interstitial edema in the lungs, gastrointestinal tract, and peripheral tissues, which disrupts epithelial and endothelial barriers and facilitates microbial translocation [11,12,13,14,15].

Neurohormonal activation, including sympathetic overdrive and RAAS up-regulation, further contributes to immune dysregulation by altering lymphocyte proliferation, macrophage function, and cytokine signaling. Chronic low-grade inflammation, a hallmark of advanced heart failure, leads to immune exhaustion and endothelial injury, amplifying susceptibility to infection. These mechanisms are frequently compounded by common comorbidities in patients with reduced LVEF, such as diabetes, obesity, and renal dysfunction, which independently impair host defense and synergistically increase infection risk [16].

Beyond hemodynamic impairment, accumulating evidence shows that therapies targeting neurohormonal and inflammatory pathways can partially reverse this state of systemic vulnerability. Angiotensin receptor–neprilysin inhibitors have been shown to improve myocardial remodeling, endothelial function, and systemic homeostasis, underscoring the close relationship between cardiac dysfunction and multisystem biological regulation [17,18,19]. Similarly, structured cardiac rehabilitation programs in patients with reduced LVEF have demonstrated beneficial effects on functional capacity, autonomic balance, endothelial performance, and inflammatory profiles, reinforcing the concept that systolic dysfunction is accompanied by potentially modifiable systemic alterations [20,21].

Together, these observations support the hypothesis that reduced LVEF identifies a biological phenotype characterized by impaired immune resilience and compromised barrier integrity rather than an isolated cardiac mechanical deficit. This multisystem vulnerability provides a plausible mechanistic framework for the increased susceptibility to HAIs observed in patients with systolic dysfunction. In this context, LVEF should be regarded not only as a marker of cardiac severity but also as an indicator of global physiological fragility that amplifies the impact of hospital-related exposures, particularly invasive devices and prolonged hospitalization.

### 4.2. Invasive Devices as Mediators of Infection Risk

Our findings strongly support the central role of invasive devices as mediators between cardiac dysfunction and infection risk. Patients with reduced LVEF had significantly higher exposure to central venous catheters, urinary catheters, pleural drains, CPAP, and endotracheal intubation, and were more frequently subjected to multiple invasive procedures. This reflects the greater hemodynamic instability and respiratory compromise of this population, which necessitate closer monitoring and more aggressive supportive care.

Although reduced LVEF itself did not remain an independent predictor in the multivariable model, device-associated risk remained dominant, particularly for urinary tract infections. The fact that the difference in overall HAI incidence was numerically driven by UTIs highlights the importance of urinary catheter management as a critical infection-control target in patients with systolic dysfunction. The association with CPAP use should be interpreted primarily as a marker of advanced cardiopulmonary compromise and increased intensity of care rather than as a direct causal effect of non-invasive ventilation itself. These findings reinforce the concept that infection prevention in CCU settings is not only a matter of antimicrobial stewardship, but also of device stewardship, where minimizing unnecessary catheterization and shortening device duration may yield substantial clinical benefits.

### 4.3. Colonization Patterns and Early Infectious Trajectory

Contrary to our initial hypothesis, colonization at admission did not differ significantly between patients with reduced and preserved LVEF and did not emerge as an independent predictor of HAIs in the present analysis. However, this absence of statistical association should be interpreted with caution. Given the limited number of HAI events and the resulting restricted statistical power, our findings do not allow us to definitively exclude a potential biological or clinical contribution of baseline colonization to subsequent infection risk. Rather than indicating a lack of effect, the results suggest that no measurable association could be demonstrated within the constraints of this cohort. At the same time, the data point toward a multifactorial trajectory in which host vulnerability and exposure to invasive procedures likely play a substantial role in shaping infection risk during hospitalization. In this context, colonization may act less as an isolated determinant and more as one component within a broader interaction between patient-related and care-related factors.

Nevertheless, the presence of colonization in a substantial proportion of CCU patients remains clinically relevant. In the context of frequent device use, compromised tissue barriers, and prolonged hospitalization, colonization can represent a latent microbial reservoir with the potential to become clinically significant. Therefore, even in the absence of statistically significant intergroup differences, admission screening retains practical value for guiding isolation precautions, informing empirical antibiotic choices, and supporting targeted surveillance strategies, particularly in clinically vulnerable subgroups such as patients with reduced LVEF.

### 4.4. Clinical Implications for Infection Control and Nursing Practice

From an infection-control perspective, our findings support the integration of LVEF into early risk stratification upon CCU admission. LVEF is an objective, readily available, and universally measured parameter that can immediately identify patients who are more likely to require invasive devices and prolonged hospitalization. Using LVEF as a vulnerability marker may help prioritize preventive resources, especially in high-demand CCU environments.

Patients with reduced LVEF may benefit from intensified preventive interventions, including stricter catheter-care bundles, daily reassessment of device necessity, reinforced hand hygiene and skin care protocols, enhanced respiratory and oral hygiene, and closer surveillance for early signs of infection. Because nursing teams play a pivotal role in device management and infection prevention, incorporating LVEF into routine nursing risk assessments may represent a potential avenue for future investigation and prospective validation.

### 4.5. Relation to Existing Literature

Most existing HAI prediction models in ICU and CCU populations focus on comorbidities, severity scores, inflammatory biomarkers, or exposure to invasive devices, while cardiac functional parameters are rarely considered [22,23,24,25]. Our study adds to the literature by demonstrating that LVEF has clear clinical relevance as a marker of infection vulnerability, even if it does not act as an independent predictor after adjustment. These findings bridge cardiology and infection-control perspectives by showing how cardiac dysfunction shapes the context in which classical infection risk factors operate.

Furthermore, the independent role of length of stay observed in our model supports previous evidence that prolonged hospitalization is a powerful driver of infection risk. Given that patients with reduced LVEF had significantly longer CCU stays, our results suggest an indirect pathway by which systolic dysfunction contributes to infection risk through prolonged exposure to the hospital environment and invasive care.

### 4.6. Strengths and Limitations

The strengths of this study include the large, consecutive cohort design, which minimizes selection bias and reflects real-world CCU practice, as well as the strict microbiological definition of HAIs. The detailed documentation of invasive devices and colonization patterns allows for mechanistic interpretation of infection pathways. The use of LVEF, an objective and standardized parameter, enhances reproducibility and clinical applicability.

Several limitations should be acknowledged. The retrospective design carries an inherent risk of unmeasured confounding and incomplete data capture. As a single-center study conducted over a limited time frame, external validity may be restricted. LVEF measurements obtained during acute illness may fluctuate and could be influenced by transient hemodynamic changes. The relatively low number of HAIs, although reflecting real-world incidence in our CCU, limits statistical power and the stability of multivariable analyses. In addition, the heterogeneity of admission diagnoses in this real-world CCU cohort may have attenuated the independent statistical effect of LVEF, as different cardiovascular conditions are associated with variable baseline risks of invasive procedures and infection. Diagnosis-specific stratified analyses were not feasible due to the limited number of events. Consequently, the logistic regression model should be regarded as exploratory rather than fully predictive. The identified associations require confirmation in larger cohorts, and the reported effect sizes should be interpreted with caution given the limited number of events.

Baseline pharmacological treatment was not systematically recorded, as the study focused primarily on cardiac functional status and care-related exposures. Although medication profiles in this specialized CCU population were generally homogeneous and cardiology-oriented, their potential influence on infection risk cannot be fully excluded.

The overall incidence of HAIs observed in our cohort was relatively low compared with rates reported in many CCU and ICU settings. This is largely explained by our strict case definition, which required microbiological confirmation and excluded clinically suspected infections without microbiological documentation. While this approach increases diagnostic specificity, it likely underestimates the true clinical burden of HAIs and may have reduced the number of events available for statistical modeling. It also increases the risk of model instability, and restricts the precision of effect size estimates. Although a formal calibration assessment was performed, the relatively low number of events limits the stability and generalizability of the model. Therefore, the multivariable analysis should still be interpreted as exploratory and hypothesis-generating rather than as a validated predictive tool.

### 4.7. Future Directions

Future studies should aim to develop and validate predictive models that integrate LVEF with device exposure, colonization status, comorbidity burden, and dynamic clinical parameters. A pragmatic bedside risk score derived from these variables could support personalized infection-prevention strategies and optimize resource allocation in CCUs. Multicenter studies are needed to confirm the generalizability of our findings and to evaluate whether LVEF-informed preventive interventions can reduce infection rates, length of stay, and overall morbidity in patients with advanced cardiac dysfunction.

In summary, reduced LVEF should not be viewed as a direct cause of HAIs, but as a powerful marker of clinical vulnerability. By identifying patients who are more likely to accumulate invasive exposures and prolonged hospitalization, LVEF provides a valuable opportunity for early, targeted, and nursing-driven infection-prevention strategies in CCUs.

## 5. Conclusions

In this large cohort of consecutively admitted CCU patients, reduced LVEF was associated with a significantly higher incidence of HAIs. Patients with impaired systolic function tended to exhibit a vulnerability profile characterized by more frequent and complex exposure to invasive devices and longer durations of hospitalization. These findings suggest that reduced LVEF may be interpreted as a marker of increased clinical vulnerability rather than as an independent causal determinant of infection.

Because LVEF is routinely assessed in patients admitted with acute cardiac conditions, it represents an easily accessible and objective parameter that may assist in the early identification of clinically vulnerable subgroups. However, these findings should be interpreted cautiously, as the multivariable analysis was exploratory and limited by the low number of events. Rather than supporting immediate changes in clinical or nursing protocols, our results suggest that integrating cardiac functional status into infection-risk stratification frameworks may be a promising area for future prospective research. Further multicenter studies are required to determine whether LVEF-guided preventive strategies can translate into measurable reductions in infection incidence and adverse clinical outcomes.

Our results also highlight the multidimensional interaction between physiological impairment, comorbidity burden, and care complexity in shaping infection trajectories in CCU patients. The observed association between systolic dysfunction and HAIs suggests that cardiac functional status may represent a potentially relevant variable for future infection-risk prediction models, although this hypothesis requires validation in larger, prospective cohorts before any practical application can be considered.

Further research is warranted to validate these observations in prospective and multicenter settings and to determine whether LVEF-guided preventive strategies can translate into meaningful reductions in infection incidence, length of stay, and adverse clinical outcomes. Building on the current findings, the future development and validation of a simple bedside risk score incorporating LVEF and other key predictors could represent a potential avenue for improving individualized infection-risk assessment in high-dependency CCUs.

## Figures and Tables

**Table 1:** Comparative baseline characteristics of the study population according to LVEF category.

Variable	LVEF < 40% (*n* = 235)	LVEF ≥ 40% (*n* = 635)
Age, years	65 (IQR 57.00–74.00)	66 (IQR 54.00–74.00)
Male, sex	165 (70.21%)	414 (65.19%)
Emergency admission	205 (87.23%)	534 (84.09%)
Length of CCU stay, days	5 (IQR 3.00–8.00)	4 (IQR 3.00–5.00)
Diabetes mellitus	65 (27.65%)	124 (19.52%)
Congestive heart failure	23 (9.78%)	28 (4.40%)
Admission infection	10 (4.25%)	27 (4.25%)
Malignancy	11 (4.68%)	15 (2.36%)

LVEF- left ventricle ejection fraction; IQR- medians with interquartile ranges; CCU- coronary care unit. Congestive heart failure was recorded as a pre-existing clinical diagnosis documented in the medical records and does not necessarily reflect the acute reason for CCU admission or the echocardiographic LVEF measured during hospitalization.

**Table 2:** Exposure to invasive devices according to LVEF category.

Invasive Device	LVEF < 40% (*n* = 235)	LVEF ≥ 40% (*n* = 635)	OR (95% CI)	*p*-Value
Peripheral venous catheter	233 (99.14%)	626 (98.58%)	1.67 (0.35–7.80)	0.736
Central venous catheter	49 (20.85%)	25 (3.93%)	6.42 (3.86–10.69)	<0.001
Arterial line	178 (75.74%)	446 (70.23%)	1.32 (0.93–1.86)	0.127
Urinary catheter	132 (56.17%)	171 (26.92%)	3.47 (2.54–4.74)	<0.001
CPAP	33 (14.04%)	14 (2.20%)	7.24 (3.80–13.81)	<0.001
Endotracheal intubation	53 (22.55%)	24 (3.77%)	7.41 (4.45–12.34)	<0.001
Pleural catheter	9 (3.82%)	7 (1.10%)	3.57 (1.31–9.70)	<0.001
Temporary pacemaker	5 (2.13%)	5 (0.79%)	2.73 (0.78–9.54)	0.145
Permanent pacemaker	12 (5.10%)	119 (18.74%)	0.23 (0.12–0.43)	<0.001
≥3 invasive devices	130 (55.31%)	238 (37.48%)	2.06 (1.52–2.79)	<0.001

CPAP- continuous positive airway pressure. Data are presented as *n* (%). ORs were calculated for LVEF < 40% versus LVEF ≥ 40%. *p*-values were obtained using the chi-square test or Fisher’s exact test, as appropriate.

**Table 3:** Multivariable logistic regression analysis for independent predictors of HAIs.

Variable	B	Exp(B)	95% CI for Exp(B)	*p*-Value
Admission infection	1.95	7.05	2.08–23.88	0.002
Malignancy	2.09	8.11	2.18–30.21	0.002
CPAP use	1.46	4.34	1.30–14.45	0.017
Length of CCU stay ≥5 days	1.72	5.62	1.21–26.08	0.027

Nagelkerke R^2^ = 0.247.

## Data Availability

The original contributions presented in this study are included in the article. Further inquiries can be directed to the corresponding author.

## Supplementary Materials

The following supporting information can be downloaded at: https://www.mdpi.com/article/10.3390/jcm15051789/s1. Table S1: Distribution of primary admission diagnoses according to LVEF category; Table S2: Colonization patterns at admission according to LVEF category; Table S3: Univariate analysis of factors associated with HAIs in CCU patients.

## Abbreviations

The following abbreviations are used in this manuscript:

CCU	Coronary Care Unit
HAI	Healthcare-Associated Infection
LVEF	Left Ventricular Ejection Fraction
CDC	Centers for Disease Control and Prevention
NHSN	National Healthcare Safety Network
OR	Odds Ratio
CI	Confidence Interval
CPAP	Continuous Positive Airway Pressure
CVC	Central Venous Catheter
PVC	Peripheral Venous Catheter
UTI	Urinary Tract Infection
ICU	Intensive Care Unit
LOS	Length of Stay
IQR	Interquartile Range
SD	Standard Deviation
AMI	Acute Myocardial Infarction
CHF	Congestive Heart Failure
HFrEF	Heart Failure with Reduced Ejection Fraction
MDR	Multidrug-Resistant
RAAS	Renin–Angiotensin–Aldosterone System
CCU LOS	Length of stay in Coronary Care Unit
