# Supplementary material for: Reduced Left Ventricular Ejection Fraction as a Marker of Vulnerability to Healthcare-Associated Infections in Coronary Care Unit Patients: A Single-Centre Cohort Study
Source: J Clin Med. 2026 Feb 27;15(5):1789. doi: 10.3390/jcm15051789 (PMC12986494; doi:10.3390/jcm15051789)
Supplement: Supplementary file 1 [file jcm-15-01789-s001.zip › jcm-4143573-supplementary.pdf]

**Table S1:** Distribution of primary admission diagnoses according to LVEF category.

| Primary diagnosis | LVEF <40% (n= 235) | LVEF ≥40% (n= 635) | p-value |
|---|---|---|---|
| Unstable angina | 6 (2.55%) | 57 (8.97%) | <0.001 |
| Stable angina | 1 (0.43%) | 8 (1.26%) | 0.45 |
| Carotid disease | 0 (0%) | 5 (0.79%) | 0.33 |
| Dilated cardiomyopathy | 18 (7.66%) | 12 (1.89%) | 0.20 |
| Endocarditis | 0 (0%) | 2 (0.31%) | 1.00 |
| Hypertension | 4 (1.70%) | 6 (0.94%) | 0.47 |
| Congestive heart failure | 23 (9.78%) | 28 (4.40%) | 0.005 |
| Acute myocardial infarction | 158 (67.23%) | 351 (55.27%) | 0.001 |
| Pericarditis | 2 (0.85%) | 1 (0.16%) | 0.179 |
| Syncope | 1 (0.43%) | 4 (0.63%) | 1.00 |
| Cardiogenic shock | 4 (1.70%) | 0 (0%) | 0.005 |
| Pulmonary thromboembolism | 0 (0%) | 3 (0.47%) | 0.56 |
| Conduction disorders | 4 (1.70%) | 120 (18.89%) | <0.001 |
| Rhythm disorders | 14 (5.96%) | 31 (4.88%) | 0.49 |
| Valvulopathy | 0 (0%) | 7 (1.10%) | 0.19 |

1. Data are presented as n (%). P-values refer to comparisons between LVEF categories using the chi-square or Fisher's exact test, as appropriate.

**Table S2.** Colonization patterns at admission according to LVEF category.

| Variable | LVEF <40% (n= 229) | LVEF ≥40% (n= 583) | OR (95% CI) | p-value |
|---|---|---|---|---|
| Positive colonization (any site) | 35 (15.28%) | 102 (17.49%) | 0.85 (0.55-1.29) | 0.468 |
| Nasal colonization | 22 (9.60%) | 76 (13.03%) | 0.70 (0.42-1.17) | 0.18 |
| Pharyngeal colonization | 2 (0.87%) | 2 (0.34%) | 2.55 (0.35-18.27) | 0.31 |
| Inguinal colonization | 0 (0%) | 3 (0.51%) | 0 (undefined) | 0.56 |
| Urinary colonization | 11 (4.80%) | 21 (3.60%) | 1.35 (0.64-2.84) | 0.42 |

2. Data are presented as n (%). ORs are calculated for LVEF <40% versus LVEF ≥40%. P-values were obtained using the chi-square test or Fisher's exact test, as appropriate.

**Table S3.** Univariate analysis of factors associated with HAIs in CCU patients.

| Variable | HAI present | HAI absent | OR (95% CI) | p-value |
|---|---|---|---|---|
| Sex (female) | 6/291 (2.06%) | 10/579 (1.73%) | 1.19 (0.43–3.32) | 0.79 |
| Environment (urban) | 10/483 (2.07%) | 6/387 (1.55%) | 1.34 (0.48–3.72) | 0.62 |
| Emergency admission | 12/739 (1.62%) | 4/131 (3.05%) | 0.52 (0.16–1.65) | 0.28 |
| LVEF <40% | 9/235 (3.82%) | 7/635 (1.10%) | 3.57 (1.31–9.70) | 0.018 |
| Diabetes mellitus | 7/189 (3.70%) | 9/681 (1.32%) | 2.87 (1.05–7.81) | 0.058 |

| Variable | HAI present | HAI absent | OR (95% CI) | p-value |
|---|---|---|---|---|
| Positive colonization (any site) | 4/137 (1.78%) | 12/675 (2.92%) | 1.66 (0.52–5.23) | 0.327 |
| Admission infection | 5/37 (13.5%) | 11/833 (1.32%) | 11.67 (3.83–35.59) | <0.001 |
| Phlebitis | 6/137 (4.38%) | 10/733 (1.36%) | 3.31 (1.18–9.26) | 0.028 |
| Malignancy | 4/26 (15.38%) | 12/844 (1.42%) | 12.60 (3.76–42.19) | <0.001 |
| Peripheral venous catheter | 16/859 (1.86%) | 0/11 (0%) | Undefined | 1.00 |
| Central venous catheter | 4/74 (5.13%) | 12/796 (1.51%) | 3.53 (1.11–11.22) | 0.046 |
| Arterial line | 9/624 (1.44%) | 7/246 (2.85%) | 0.49 (0.18–1.35) | 0.16 |
| Urinary catheter | 10/303 (3.30%) | 6/567 (1.06%) | 3.19 (1.14–8.86) | 0.030 |
| CPAP | 5/47 (10.64%) | 11/823 (1.34%) | 8.78 (2.92–26.44) | 0.001 |
| Endotracheal intubation | 3/77 (3.90%) | 13/793 (1.64%) | 2.43 (0.67–8.72) | 0.16 |
| Pleural catheter | 1/16 (5.88%) | 15/854 (1.76%) | 3.49 (0.43–28.08) | 0.272 |
| Permanent pacemaker | 2/154 (1.30%) | 14/716 (1.96%) | 0.65 (0.14–2.93) | 0.75 |
| Surgical (pacemaker) wound | 2/127 (1.55%) | 14/743 (1.88%) | 0.82 (0.18–3.65) | 1.00 |
| Chronic wound | 1/27 (3.70%) | 15/843 (1.78%) | 2.12 (0.27–16.68) | 0.39 |
| ≥3 invasive devices | 10/368 (2.72%) | 6/502 (1.20%) | 2.30 (0.83–6.41) | 0.125 |
| Length of CCU stay ≥5 days | 14/380 (3.38%) | 2/490 (0.41%) | 9.33 (2.10–41.32) | <0.001 |

3. Data are presented as n (%). ORs were calculated using univariate logistic regression. "Undefined" indicates that ORs could not be calculated due to zero events in one of the comparison groups. P-values were obtained using the chi-square test or Fisher's exact test, as appropriate.
